# Wildlife-associated *Cryptosporidium fayeri* in Human, Australia

**DOI:** 10.3201/eid1612.100715

**Published:** 2010-12

**Authors:** Liette S. Waldron, Cristel Cheung-Kwok-Sang, Michelle L. Power

**Affiliations:** Author affiliation: Macquarie University, Sydney, New South Wales, Australia

**Keywords:** Cryptosporidium, epidemiology, human disease, cryptosporidiosis, marsupial, glycoprotein 60, zoonoses, letter

**To the Editor:** Molecular tools are essential for *Cryptosporidium* spp. identification, taxonomy, and epidemiology because of morphologic similarities between species within this genus. Molecular analyses have now identified 22 *Cryptosporidium* spp. and >40 cryptic species (i.e., genotypes) across all vertebrate classes (*1*). The myriad of potential *Cryptosporidium* spp. hosts, in conjunction with the robustness of the infectious stage (oocyst), means diverse *Cryptosporidium* spp. constantly circulate through the environment. This circulation increases the potential for disease from a diversity of contamination sources.

Human cryptosporidiosis is a global problem causing illness in young, elderly, immunocompromised, and immunocompetent persons in both industrialized and developing nations. The 2 most common etiologic agents, responsible for 90% of reported human infections, are *C. hominis* and *C. parvum* (*2**,**3*). Additional species identified as human pathogens are *C. meleagridis*, *C. canis*, *C. felis*, and the *Cryptosporidium* rabbit genotype (*4*). Each of these species was once thought to be specific for turkeys, dogs, cats, and rabbits, respectively. Incidental findings of *C. muris*, *C. andersoni*, *C. suis*, *C. hominis* monkey genotype, *C. parvum* mouse genotype, and *Cryptosporidium* cervine (W4), chipmunk I (W17), skunk, and horse genotypes have also been reported in humans (*4*). The pathogenicity of these zoonotic species and genotypes to humans remains unclear.

In July 2009, a 29-year-old woman who sought care because of prolonged gastrointestinal illness had a fecal test positive for *Cryptosporidium* spp. by the Remel ProSpecT *Giardia*/*Cryptosporidium* microplate assay (Thermo Fisher Scientific, Lenexa, KS, USA). Oocysts were purified from the specimen (*5*) and stained with the *Cryptosporidium* spp.–specific antibody CRY104 labeled with fluorescein isothiocyanate (Biotech Frontiers, North Ryde, Australia) for enumeration. A parasite load of 1.34 × 10^6^ oocysts/g feces was determined by using epifluorescence microscopy at 400× magnification.

To identify *Cryptosporidium* spp., DNA was extracted (*5*), and a diagnostic fragment of the small subunit (SSU) rRNA) was amplified (*6*). Clones were screened to identify species and determine the possibility of mixed infection. Plasmids from 50 clones were recovered and digested with the enzyme *SspI* (New England Biolabs, Beverly, MA, USA) (*6*). Two different restriction profiles were visualized. The sequence from each of the restriction types was determined; profile 1 contained *SspI* fragment sizes of 33, 109, 247, and 441 bp; profile 2 had fragments of 33, 254, and 540bp. A BLAST search (www.ncbi.nlm.nih.gov/blast) confirmed the sequences as *C. fayeri* type 1 and type 2. These 2 sequences correspond to known heterogeneity within the SSU rRNA of *C. fayeri* (*7*).

The identification of *C. fayeri* by SSU rRNA was confirmed by the sequence of the actin gene (*8*), showing 99.8% similarity to *C. fayeri* (GenBank accession no. AF112570). Further analysis at the 60-kDa glycoprotein (gp60) locus was used to determine the *Cryptosporidium* subtype family (*5*). The MQ1022 gp60 sequence was 98% similar to *C. fayeri* subtype family IVa (*9*). Analysis of the microsatellite region further characterized isolate MQ1022 to *C. fayeri* subtype IVaA9G4T1R1. The nucleotide sequences generated in this study were submitted to GenBank under accession nos. HQ008932–HQ008934.

Because the patient was imunocompetent, the disease was believed to be self-limiting, and she was lost to follow-up. The patient resided in a national forest on the east coast of New South Wales, Australia, an area where marsupials are abundant. She had frequent contact with partially domesticated marsupials. Notably, *C. fayeri* has been identified in 6 Australian marsupial species. Identification of *C. fayeri* in a human patient is a concern for water catchment authorities in the Sydney region. The main water supply for Sydney, Warragamba Dam, covers 9,050 km^2^ and is surrounded by national forest inhabited by diverse and abundant marsupials. A previous study that investigated *Cryptosporidium* spp. in a wild eastern gray kangaroo (*Macropus giganteus*) population reported a prevalence of 6.7% (*10*). Oocyst shedding ranged from 20/g feces to 2.0 × 10^6^/g feces (*10*). Subtype IVaA9G4T1R1 identified from the patient in this study has been characterized from eastern gray kangaroos in Warragamba Dam (*9*). Throughout the year, large groups of eastern gray kangaroos graze within riparian zones in the catchment. Such close proximity to the water presents a high possibility that the dam’s water is contaminated with oocysts from these animals.

The *Cryptosporidium* genus is diverse, both in species and suitable hosts. The mechanisms of host specificity remain unknown, but the frequency of *Cryptosporidium* spp. crossing the host barrier and becoming zoonoses is increasing. This increase indicates that *Cryptosporidium* spp. host specificity is not as stringent as previously thought.
